# Epidemiological assessment of cassava mosaic disease in Burkina Faso

**DOI:** 10.1111/ppa.13459

**Published:** 2021-09-09

**Authors:** Monique Soro, Fidèle Tiendrébéogo, Justin S. Pita, Edwig T. Traoré, Koussao Somé, Ezechiel B. Tibiri, James B. Néya, J. Musembi Mutuku, Jacques Simporé, Daouda Koné

**Affiliations:** ^1^ Central and West African Virus Epidemiology (WAVE) Pôle scientifique et d’innovation de Bingerville Université Félix Houphouët‐Boigny (UFHB) Bingerville Ivory Coast; ^2^ Laboratoire de Biotechnologie, Agriculture et Valorisation des Ressources Biologiques UFR Biosciences Université Félix Houphouët‐Boigny Abidjan Ivory Coast; ^3^ Laboratoire de Virologie et de Biotechnologies Végétales Institut de l’Environnement et de Recherches Agricoles (INERA) Ouagadougou Burkina Faso; ^4^ Laboratoire Mixte International Patho‐Bios IRD‐INERA Ouagadougou Burkina Faso; ^5^ Laboratoire de Biologie Moléculaire et de Génétique (LABIOGENE) Université Joseph Ki‐Zerbo Ouagadougou Burkina Faso; ^6^ Laboratoire de Génétique et de Biotechnologies Végétales Institut de l’Environnement et de Recherches Agricoles (INERA) Ouagadougou Burkina Faso; ^7^ Centre d’Excellence Africain sur le Changement Climatique, la Biodiversité et l’Agriculture Durable (WASCAL/CEA‐CCBAD, Université Félix Houphouët‐Boigny) PSI‐Université Félix Houphouët‐Boigny Abidjan Ivory Coast

**Keywords:** African cassava mosaic virus (ACMV), cassava mosaic geminiviruses, East African cassava mosaic Cameroon virus (EACMCMV), geminiviruses characterization, geminiviruses distribution

## Abstract

Surveys were conducted in 2016 and 2017 across the main cassava‐growing regions of Burkina Faso to assess the status of cassava mosaic disease (CMD) and to determine the virus strains causing the disease, using field observation and phylogenetic analysis. CMD incidence varied between regions and across years but was lowest in Hauts‐Bassins (6.0%, 2016 and 5.4%, 2017) and highest in Centre‐Sud (18.5%, 2016) and in Boucle du Mouhoun (51.7%, 2017). The lowest CMD severity was found in Est region (2.0) for both years and the highest in Sud‐Ouest region (3.3, 2016) and Centre‐Sud region (2.8, 2017). The CMD infection was primarily associated with contaminated cuttings in all regions except in Hauts‐Bassins, where whitefly‐borne infection was higher than cuttings‐borne infection in 2016. PCR screening of 687 samples coupled with sequence analysis revealed the presence of African cassava mosaic‐like (ACMV‐like) viruses and East African cassava mosaic‐like (EACMV‐like) viruses as single infections at 79.5% and 1.1%, respectively. Co‐infections of ACMV‐like and EACMV‐like viruses were detected in 19.4% of the tested samples. In addition, 86.7% of the samples positive for EACMV‐like virus were found to be positive for East African cassava mosaic Cameroon virus (EACMCMV). Phylogenetic analysis revealed the segregation of cassava mosaic geminiviruses (CMGs) from Burkina Faso into three clades specific to ACMV, African cassava mosaic Burkina Faso virus (ACMBFV), and EACMCMV, confirming the presence of these viruses. The results of this study show that EACMCMV occurrence may be more prevalent in Burkina Faso than previously thought.

## Graphical abstract

Cassava mosaic disease is widespread in Africa. In Burkina Faso, we assessed the main epidemiological parameters and characterized the major viruses associated with the disease.

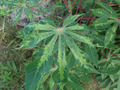

## 1 INTRODUCTION

Cassava (*Manihot esculenta*, Euphorbiaceae), which originates from Latin America, is a major source of food for more than 700 million people in tropical and subtropical developing countries and enhances food security in these countries (Ntawuruhunga et al., 2013; Patil & Fauquet, 2009; Saediman et al., 2016). Cassava is a staple food crop in the sub‐Saharan region of Africa and consequently a source of income for many processors and traders (Ntawuruhunga et al., 2013). The high calorie yield per hectare (250 kcal/ha/day), drought tolerance, hardiness in stressful environments, and flexibility of harvesting time are the major advantages of this crop compared to many other crops (Byju & Suja, 2020; El‐Sharkawy, 2004; Pushpalatha & Gangadharan, 2020). In Burkina Faso, cassava was introduced by farmers decades ago from Ghana and Ivory Coast (Côte d’Ivoire) (Guira et al., 2017). It has long been cultivated around vegetable gardens for domestic consumption. Formerly considered as a neglected crop, cassava has become a cash crop since the formal introduction of improved varieties from IITA in 2003 (Dabiré & Belem, 2003).

In Africa, cassava production is negatively affected by two main viral diseases: cassava brown streak disease (CBSD) and cassava mosaic disease (CMD). CMD is a major constraint to cassava production, which causes tuber yield losses estimated at $2.7 billion annually (Patil & Fauquet, 2009). CMD is caused by distinct cassava mosaic geminiviruses (CMGs) (family *Geminiviridae*, genus *Begomovirus*) and naturally transmitted by the whitefly *Bemisia tabaci* (Hemiptera: Aleyrodidae) (Ally et al., 2019; Legg et al., 2002; MacFadyen et al., 2018). CMD is also widely disseminated by infected stem cuttings, used for vegetative propagation (Bock & Woods, 1983; Fondong et al., 2000). CMD is endemic in Africa, with nine distinct CMG species officially recognized by the International Committee on Taxonomy of Viruses (ICTV; https://talk.ictvonline.org/ictv‐reports/ictv_online_report/ssdna‐viruses/w/geminiviridae/479/member‐species‐begomovirus): African cassava mosaic Burkina Faso virus (ACMBFV; Tiendrébéogo et al., 2012), African cassava mosaic virus (ACMV; Stanley & Gay, 1983), East African cassava mosaic Cameroon virus (EACMCMV; Fondong et al., 2000), East African cassava mosaic Kenya virus (EACMKV; Bull et al., 2006), East African cassava mosaic Malawi virus (EACMMV; Zhou et al., 1998), East African cassava mosaic virus (EACMV; Pita, Fondong, Sangaré, Otim‐Nape, et al., 2001), East African cassava mosaic Zanzibar virus (EACMZV; Maruthi et al., 2004), cassava mosaic Madagascar virus (CMMGV), and South African cassava mosaic virus (SACMV; Berrie et al., 2001).

In West Africa, the presence of ACMV and EACMV was reported in Ivory Coast (Pita, Fondong, Sangaré, Kokora, et al., 2001; Toualy et al., 2014), Ghana (Torkpo et al., 2017), and Nigeria (Abubakar et al., 2019; Ariyo et al., 2005; Eni et al., 2021; Ogbe et al., 2006). The presence of EACMCMV was also reported in Ivory Coast and Nigeria (Ariyo et al., 2005; Eni et al., 2021; Pita, Fondong, Sangaré, Kokora, et al., 2001). In previous studies, the presence of cassava mosaic disease (CMD) has been reported in some localities in Burkina Faso. Indeed, the presence of ACMV was reported in 1995 using the triple antibody sandwich‐ELISA method with cross‐reacting monoclonal antibodies to ACMV (Konaté et al., 1995). The molecular features of an ACMV‐like virus (ACMBFV, whose Rep protein gene and intergenic region differ from ACMV) was described and the presence of EACMV‐UG variant was reported around Ouagadougou (Tiendrébéogo et al., 2009, 2012). Since then, the real status of CMD and its epidemiological parameters such as the incidence and severity of the disease, the whitefly abundance, and the mode of infection remain unclear. To overcome this knowledge gap, we conducted for the first time georeferenced surveys in the main cassava production areas in Burkina Faso.

## 2 MATERIALS AND METHODS

### 2.1 Cassava mosaic disease status assessment

**FIGURE 1:**
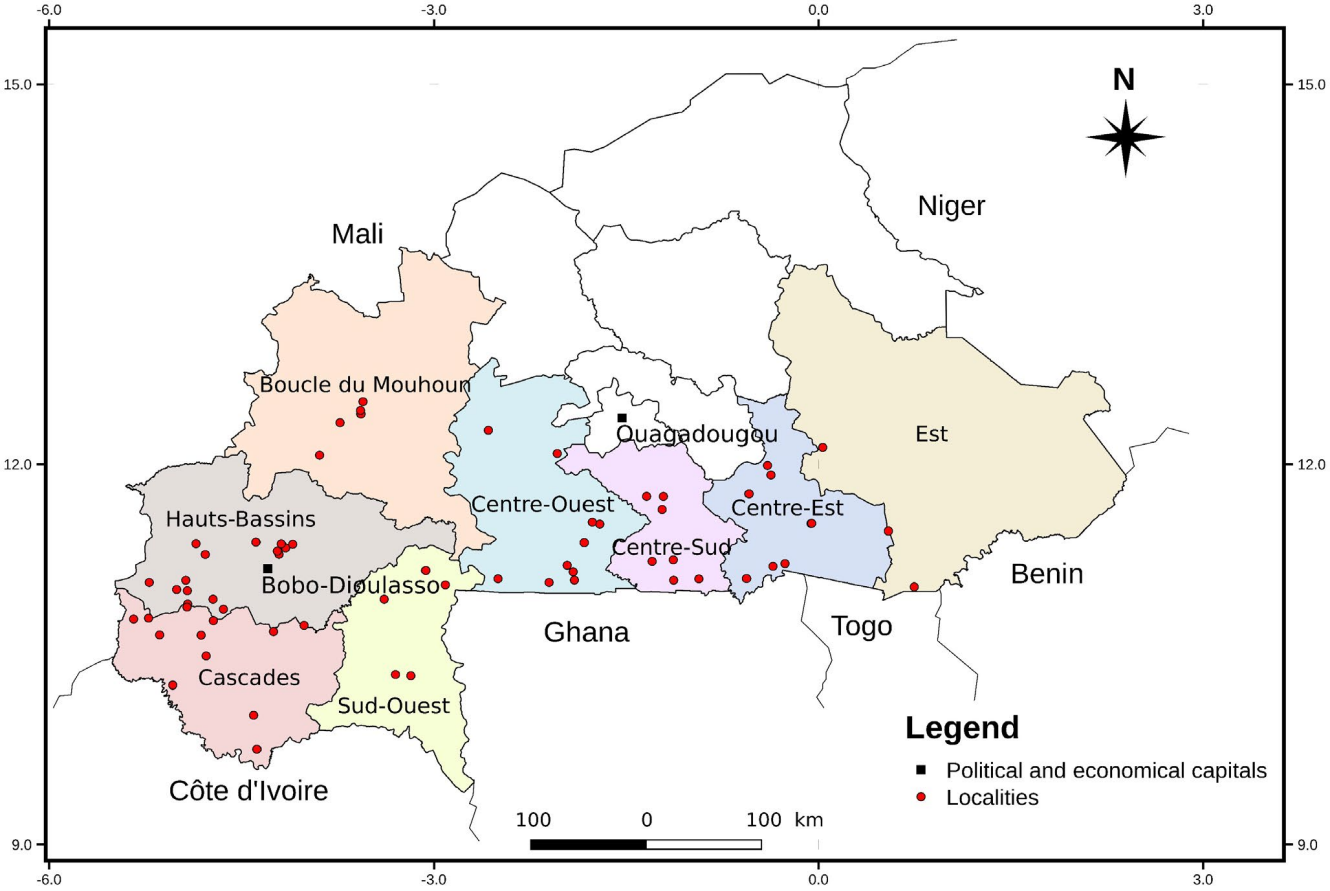
Map of Burkina Faso showing the regions and localities where surveys were done in 2016 and 2017 [Colour figure can be viewed at wileyonlinelibrary.com]

Surveys were conducted in 2016 and 2017 in eight major cassava‐growing regions of Burkina Faso (Figure 1). The number of fields sampled within a region depended on the number of cassava‐growing localities and the availability of cassava fields at 3–6 months after planting (MAP). Our field sampling protocol was a modification of one previously described (Sseruwagi et al., 2004) and has been adopted by 10 countries in Central and West Africa to harmonize efforts at surveillance and monitoring of these transboundary pathogens of high economic importance. Briefly, in each field, 30 cassava plants were assessed randomly along two diagonals to form an “X” pattern. Then each selected plant was assessed visually for the presence or absence of CMD symptoms (leaf mosaic, leaf distortion, and stunted growth) and the number of whiteflies settling on the leaves, and if infected, we determined whether the mode of infection was through cuttings or whitefly transmission. The whitefly population was estimated by counting the number of whiteflies on the top five fully expanded leaves. The mode of infection in each plant was determined based on the location of the leaves with symptoms as previously described by Sseruwagi et al. (2004). According to these authors, from 3 to 6 MAP it is possible to distinguish between cutting‐borne and whitefly‐borne infections. Symptoms appearing only on upper leaves were taken to have resulted from whitefly‐transmitted infection, whereas plants that showed symptoms either only on the lower leaves or on all leaves were taken as having been infected through cassava cuttings. CMD symptom severity was recorded using a scale from 1 (no symptoms) to 5 (very severe symptoms) (Terry, 1975). We acknowledge that the severity level depends on the variety, climate, crop management, and mainly the time at which the infection occurred. To minimize the effects of these variables on our data, we sampled fields within the same locations that were within the 3–6 MAP age. The CMD incidence was calculated as the percentage of plants with symptoms in relation to the number of plants assessed.

A total of 237 leaf samples from 212 plants with symptoms and 25 without symptoms in 2016, and 450 leaf samples from 240 plants with symptoms and 210 without symptoms in 2017 were collected for laboratory analysis using PCR and Sanger sequencing. The global positioning system (GPS) coordinates were recorded for each field.

### 2.2 Molecular characterization of CMGs

Total DNA was extracted from cassava leaves using the CTAB protocol as previously described (Permingeat et al., 1998). The concentration of DNA in each sample was determined using a NanoDrop 2000 spectrophotometer (Thermo Fisher Scientific) and adjusted to 150 ng/μl. We previously discovered that the most problematic CMGs in smallholder cassava production in Burkina Faso were ACMV and a variant of the East African cassava mosaic virus (EACMV), the EACMV‐Uganda variant (Tiendrébéogo et al., 2009). Because the current status of the incidence and severity of these two CMD‐causing viruses is unknown in Burkina Faso, we surveyed the whole country and focused our surveys on ACMV and EACMV. The extracted DNA was subjected to PCR using the specific primers listed in Table 1 for the detection of ACMV‐like virus (JSP001/JSP002) and EACMV‐like virus (JSP001/JSP003). The samples positive for EACMV‐like virus were subjected to another round of PCR using specific primers for the detection of EACMCMV (VNF031/VNF032; Table 1). The PCR mix was prepared in a final volume of 25 μl using 20.9 μl of molecular biology grade water, 2.5 µl of 10× reaction buffer, 0.5 µl of 10 mM dNTPs, 0.5 µl of 10 µM of each primer, 0.1 µl of 5 U/µl of Maximo *Taq* DNA polymerase (GeneON), and 150 ng DNA template of each sample. The DNA amplification was carried out in a SimpliAmp thermal cycler (Life Technologies Holdings Pte Ltd). The PCR temperature profile was set at 94°C for 4 or 5 min for initial denaturation, followed by 35 cycles of amplification at 94°C for 45 or 60 s, 55°C for 45 or 60 s, and 72°C for 55 or 60 s (depending on primers). The final elongation step was performed at 72°C for 7 or 10 min. PCR‐amplified products were subjected to 1% agarose gel electrophoresis, stained with ethidium bromide. The electrophoresis was performed at 100 V and the gel was visualized using a Compact Digimage System, UVDI series (MS major science). PCR products of 40 ACMV‐like positive samples (randomly selected from the regions) were directly sequenced in both forward and reverse orientations using the Sanger method at Inqaba Biotec company (South Africa) to determine their identity. PCR products of 15 EACMCMV positive samples were also subjected to sequencing in both forward and reverse orientations to confirm their identity.

**TABLE 1:** Primer pairs used for the amplification of ACMV‐like virus, EACMV‐like virus, and EACMCMV

Primer	Sequence (5′–3′)	Target region	Expected size (bp)	Virus species	Reference
JSP 001	ATGTCGAAGCGACCAGGAGAT	DNA‐A (CP)	783	ACMV‐like virus	Pita, Fondong, Sangaré, Otim‐Nape, et al. (2001)
JSP 002	TGTTTATTAATTGCCAATACT				
JSP 001	ATGTCGAAGCGACCAGGAGAT	DNA‐A (CP)	780	EACMV‐like virus	Pita, Fondong, Sangaré, Otim‐Nape, et al. (2001)
JSP 003	CCTTTATTAATTTGTCACTGC				
VNF031/F	GGATACAGATAGGGTTCCCAC	DNA‐A (AC2/AC3)	560	EACMCMV	Fondong et al. (2000)
VNF032/R	GACGAGGACAAGAATTCCAAT				

### 2.3 Statistical analysis

Data analysis was performed using the R software v. 3.6.1 (R Development Core Team). The normality of the variables was determined using the Shapiro–Wilk test. When the variable was not distributed according to the normal distribution, the generalized linear model was used. The difference in the number of whiteflies per plant between regions and the difference in the severity score of CMD between regions in the same year were assessed using the generalized linear model and Tukey's pairwise mean comparison test. The difference in the number of whiteflies per plant and the difference in the severity score of CMD between 2016 and 2017 were assessed using Wilcoxon test with continuity correction. A test of pairwise comparison of proportions was used based on a G‐test with correction of BY (Benjamini & Yekutieli, 2001) to compare the incidences of CMD between regions. The map of Burkina Faso showing the regions where surveys were done in 2016 and 2017 was developed using QGIS software v. 2.18.26 (https://qgis.org/downloads/).

### 2.4 Phylogenetic analysis

The amplicon sequences were trimmed and assembled de novo using Geneious v. 8.1.7 (Biomatters Ltd) software. Consensus sequence obtained from forward and reverse sequences for each sample was subjected to BLASTn in NCBI for preliminary species assignment and subsequently for pairwise sequence comparison (Bao et al., 2014). The sequences were aligned with representative isolates of begomoviruses using ClustalW alignment method in MEGA X software (Kumar et al., 2018). The sequences of 25 out of 40 ACMV‐like virus positive samples and six out of 15 EACMCMV positive samples were used for phylogenetic tree construction. The maximum‐likelihood (ML) method with general time reversible (GTR) model (as the best fit model for substitution pattern description) was used for phylogenetic trees construction using FastTree v. 2.1.9 (Price et al., 2010) with bootstrap replicates of 1000. The tree was visualized and edited using FigTree v. 1.4.4 (http://tree.bio.ed.ac.uk/software/figtree/).

## 3 RESULTS

### 3.1 CMD distribution in 2016 and 2017

**FIGURE 2:**
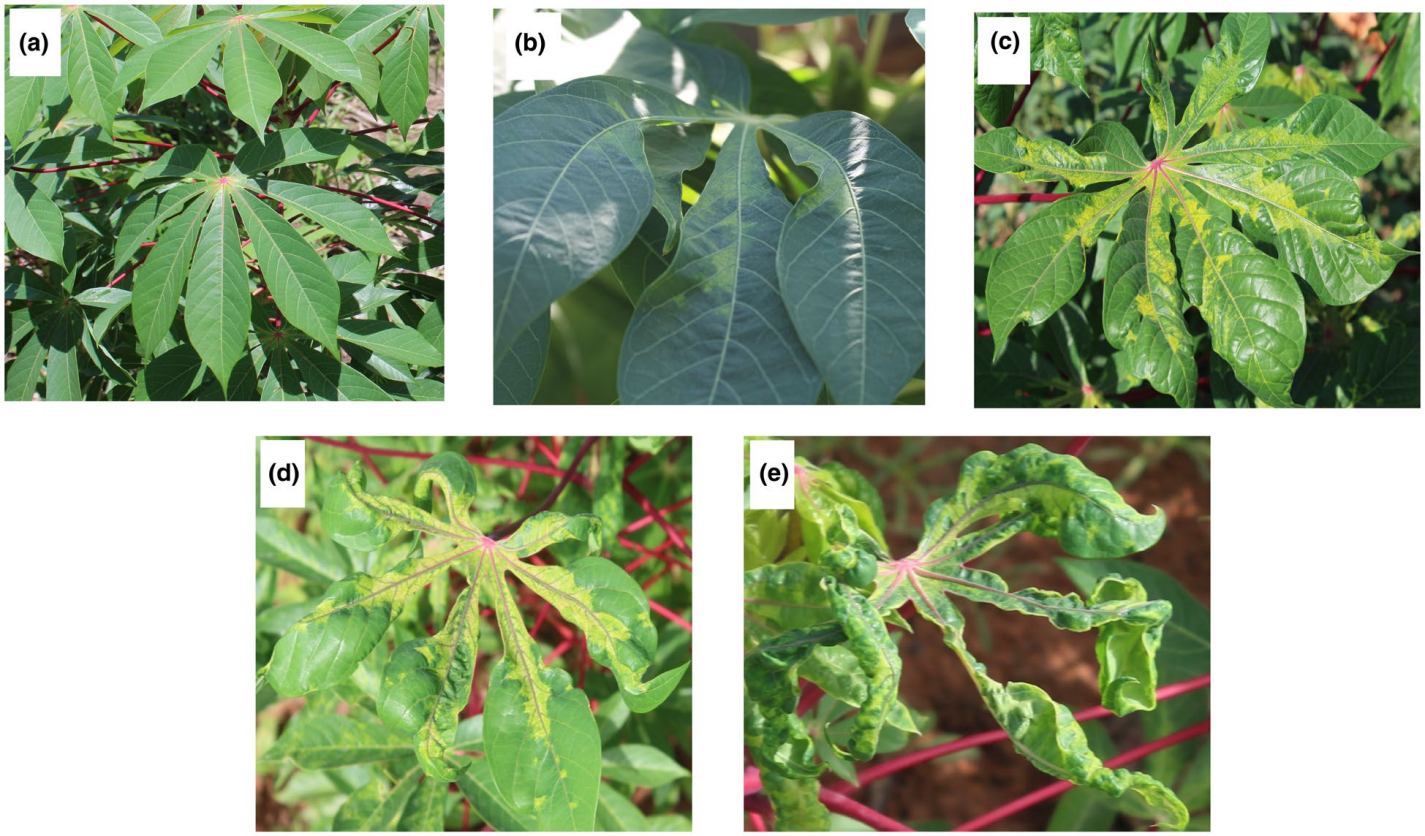
Symptoms of cassava mosaic disease observed on infected cassava plants during the surveys, using a scale from 1 (no symptoms) to 5 (very severe symptoms). (a) = 1, (b) = 2, (c) = 3, (d) = 4, (e) = 5 [Colour figure can be viewed at wileyonlinelibrary.com]

In 2016, CMD symptoms were found in 84.0% (42/50) of surveyed localities, with the lowest proportion (57.1%, 8/14) of infected fields in the region of Hauts‐Bassins. Cassava fields in 65.9% (29/44) of localities showed CMD symptoms in 2017, with the lowest proportion (45.5%, 5/11) in Centre‐Est region (Table 2). The proportion of localities where CMD‐affected cassava fields were found varied significantly between 2016 and 2017 (*p* < 0.05). Indeed, compared to 2016, no cassava fields were found to have CMD symptoms in the provinces of Nahouri (Centre‐Sud region) and Bougouriba (Sud‐Ouest region) in 2017. Typical CMD symptoms observed across farmers’ fields included distinctive leaf mosaic symptoms often associated with leaf distortion and reduction (Figure 2b–e), as well as an overall stunted appearance of the affected plants.

**TABLE 2:** Proportion of localities in Burkina Faso where typical cassava mosaic disease (CMD) symptoms were found on cassava plants in 2016 and 2017

Region	2016			2017		
	Surveyed localities	Localities with CMD	Localities with CMD (%)	Surveyed localities	Localities with CMD	Localities with CMD (%)
Boucle du Mouhoun	5	4	80.0	2	2	100.0
Cascades	11	10	91.9	7	7	100.0
Centre‐Est	1	1	100.0	11	5	45.4
Centre‐Ouest	6	6	100.0	6	5	83.3
Centre‐Sud	8	8	100.0	4	2	50.0
Est	2	2	100.0	2	2	100.0
Hauts‐Bassins	14	8	57.1	8	4	50.0
Sud‐Ouest	3	3	100.0	4	2	50.0
Total	50	42	84.0	44	29	65.9

### 3.2 Incidence and symptom severity of CMD in 2016 and 2017

**FIGURE 3:**
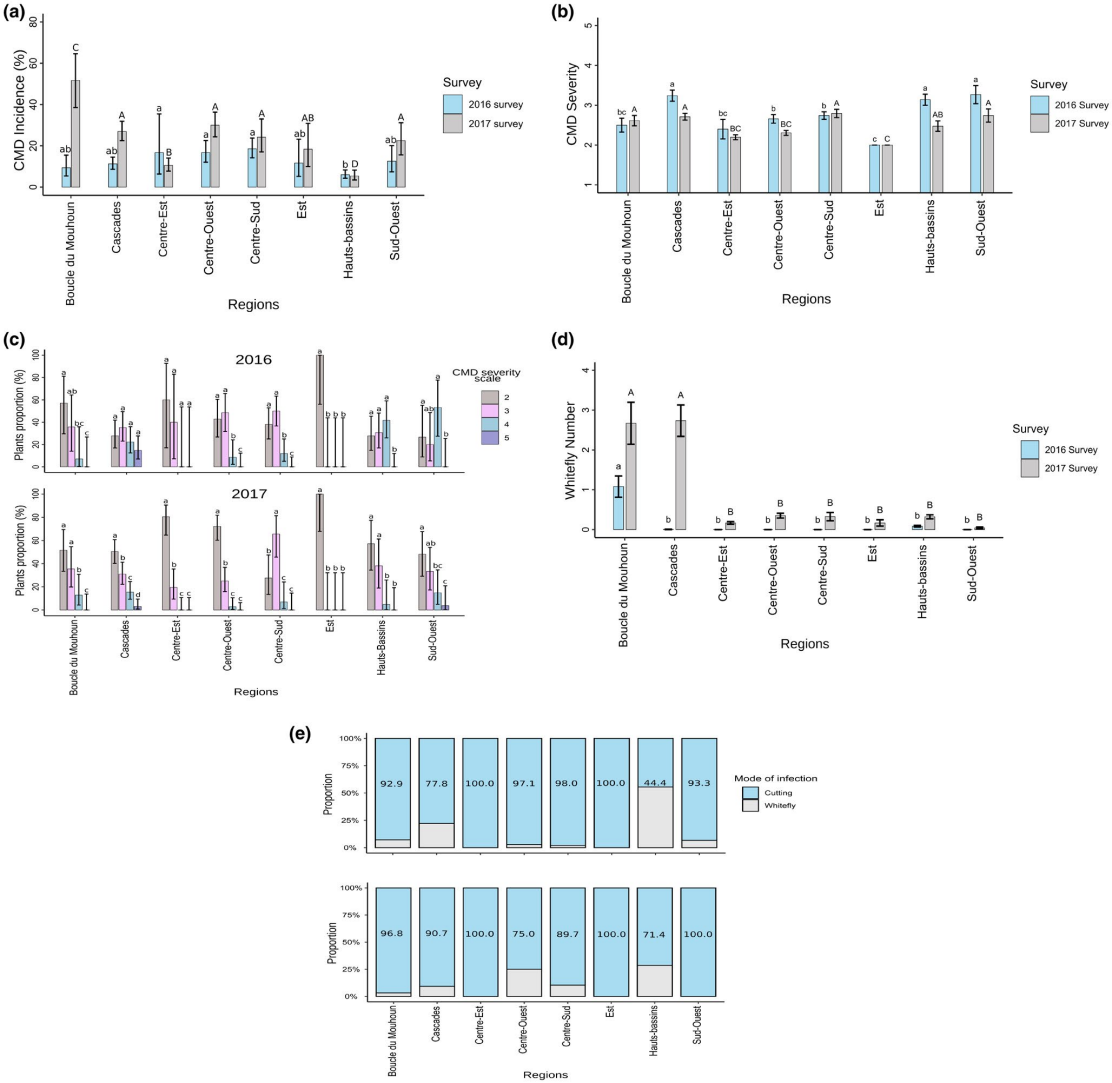
Epidemiological assessment of cassava mosaic disease (CMD) in Burkina Faso. (a) CMD incidence (percentage of plants with symptoms). (b) Severity of CMD (mean CMD severity score of plants with symptoms). (c) Proportion of plants with different CMD severity scores in 2016 and 2017. (d) Mean whitefly counts in 2016 and 2017. (e) Proportion of plants with symptoms infected by cutting or whitefly in 2016 and 2017. The bars represent the standard error. Bars sharing the same lower case letters are not significantly different between regions in 2016 and those sharing the same upper case letters are not significantly different between regions in 2017 [Colour figure can be viewed at wileyonlinelibrary.com]

The CMD incidence in 2016 varied significantly from that observed in 2017 (*p* ≤ 0.001). In 2016, the overall CMD incidence across the surveyed fields in Burkina Faso was 11.3% (216/1920) and ranged from 6.0% (36/600) in Hauts‐Bassins region to 18.5% (50/270) in Centre‐Sud region. In 2016, the difference between the lowest incidence and the highest incidence was highly significant (*p* ≤ 0.001). For the 2017 survey, the overall CMD incidence was 18.9% (329/1720). The lowest incidence in 2017 was observed in the Hauts‐Bassins region with 5.4% (21/390), whereas the Boucle du Mouhoun region was observed to have the highest incidence 51.7% (30/60, *p* ≤ 0.001; Figure 3a).

The mean CMD symptom severity score was 2.9 and the range was from 2.0 (Est region) to 3.3 (Sud‐Ouest region) in 2016. In 2017, the mean CMD symptom severity score was 2.5 with the lowest severity in Est region (2.0) and the highest severity in the Centre‐Sud region (2.8) (Figure 3b). Significant differences (*p* < 0.05) were found between CMD symptom severity scores in 2016 and 2017 in three regions (Cascades, Centre‐Ouest, and Hauts‐Bassins). In most of the regions, no significant difference was found between the proportion of plants with different CMD symptom severity scores in 2016. In 2017, the proportion of plants with CMD symptom severity score 2.0 was higher than the other symptom severity scores in most of the regions, whereas in the region of Centre‐Sud the proportion of plants with CMD symptom severity score 3 was the highest (Figure 3c).

### 3.3 Adult whitefly distribution and mode of infection

Determination of whitefly counts and distribution was conducted at the time of the CMD incidence and severity survey to ensure the parameters that might affect the epidemiology of CMD in the field were the same, and the plants were of similar age. The methods have been harmonized across 10 West and Central African countries, Sierra Leone, Ivory Coast, Burkina Faso, Ghana, Nigeria, Benin, Togo, Cameroon, Gabon, and Democratic Republic of Congo, to ensure the data is comparable. The adult whitefly counts were very low in 2016 and a similar trend was observed in 2017, with a mean of 0.1 and 0.7 per plant in 2016 and 2017, respectively. The highest mean whitefly count was observed in the region of Boucle du Mouhoun (1.08) in 2016. In 2017, the highest mean whitefly count (2.7) was observed in the regions of Boucle du Mouhoun and Cascades. In most of the regions, the mean number of whiteflies per plant was higher in 2017 than in 2016 (Figure 3d).

When the number of plants with symptoms infected through cuttings or by whitefly transmission were compared, a preponderance of cutting‐borne infections was detected in 2016 (83.3%, 180/216) and 2017 (88.8%, 292/329). The exception was Hauts‐Bassins region, where greater whitefly‐borne infections were recorded in 2016 (Figure 3e). It is likely that a number of factors including climatic conditions, infection status, or indeed cassava variety differences affected whitefly counts (Mugerwa et al., 2021). The challenge is that during our survey years, whitefly pressure was not strong enough to explain the significant differences in disease incidence observed between regions and years. Also, considering that high incidence is associated with seeding using infected cuttings, we are not able to without doubt correlate whitefly numbers with the disease incidence or severity.

### 3.4 CMGs detected by PCR in cassava leaf samples

A total of 687 cassava leaf samples were collected from 452 plants with symptoms and 235 plants without symptoms in 2016 and 2017 for PCR analysis. Among the samples having observable symptoms, 4.0% (18/452) tested negative for ACMV‐like virus and EACMV‐like virus. On the other hand, 2.1% (5/235) of symptomless samples tested positive for ACMV‐like virus. Approximately 63.9% (439/687) of collected samples tested positive for CMGs. Among the positive samples, the single ACMV‐like virus infection was by far the most frequent, accounting for 79.5% (349/439) of all infection, followed by mixed infections of ACMV‐like virus and EACMV‐like virus with 19.4% (85/439), and single infection of EACMV‐like virus with 1.1% (5/439). The single infection of ACMV‐like virus was predominant in all surveyed regions, with the highest proportion (100%) in the Centre‐Est and Est regions. The mixed infection occurred in the remaining six regions, with the highest proportions in Centre‐Sud (42.6%, 29/68) and Sud‐Ouest (46.4%, 13/28) regions. The single infection of EACMV‐like virus was found in the regions of Sud‐Ouest (3.6%, 1/28), Cascades (2.3%, 3/132) and Centre‐Sud (1.5%, 1/68) but no significant difference was found between these proportions (Table 3). Of the 90 EACMV‐like virus positive samples (single and mixed infections), 86.7% (78/90) tested positive for EACMCMV using the primer pair VNF031/VNF032.

**TABLE 3:** PCR results obtained from samples collected during 2016 and 2017 surveys in eight main cassava‐growing regions of Burkina Faso

Region	Tested samples	Positive samples	ACMV‐like virus single infection		EACMV‐like virus single infection		Mixed infection	
			*n*	%	*n*	%	*n*	%
Boucle du Mouhoun	30	26	19	73.1 ad	0	0.0	7	26.9 ab
Cascades	202	132	107	81.1 ab	3	2.3	22	16.6 bc
Centre‐Est	90	43	43	100.0 c	0	0.0	0	0.0 d
Centre‐Ouest	95	70	62	88.6 ab	0	0.0	8	11.4 bc
Centre‐Sud	84	68	38	55.9 d	1	1.5	29	42.6 a
Est	35	18	18	100.0 c	0	0.0	0	0.0 d
Hauts‐Bassins	113	54	48	88.9 ab	0	0.0	0	11.1 bc
Sud‐Ouest	38	28	14	50.0 d	1	3.6	13	46.4 a
Total	687	439	349	79.5	5	1.1	85	19.4

Note

Percentages followed by the same letters are not significantly different between regions.

### 3.5 CMGs identity confirmed by sequencing

**FIGURE 4:**
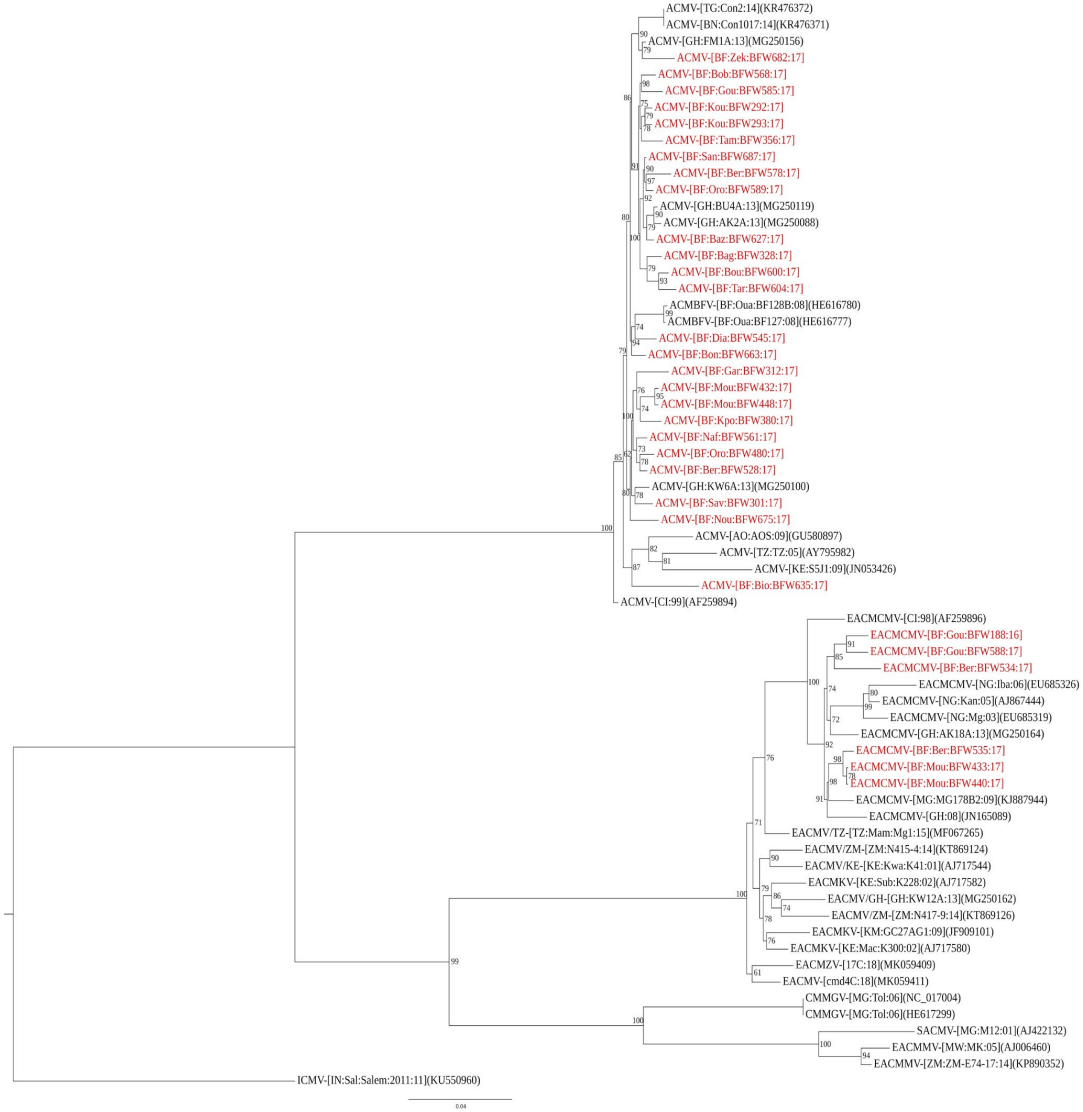
Maximum‐likelihood phylogenetic tree obtained from alignment of partial nucleotide sequences of coat protein (CP) genes of African cassava mosaic‐like viruses (ACMV‐like) and East African cassava mosaic‐like viruses (EACMV‐like). The names of the sequences characterized in this study are in red. The horizontal scale indicates the genetic distance [Colour figure can be viewed at wileyonlinelibrary.com]

A search for related sequences in the GenBank database (NCBI, BLASTN) showed that the sequences of the 40 samples that tested positive for the ACMV‐like virus were most closely related to ACMV and ACMBFV. Indeed, they shared the highest nucleotide identity (98%–99%) with ACMV isolates from Ghana (MG250119, MG250156, MG250088), Ivory Coast (AF259894), Burkina Faso (FM877473), and Nigeria (MH251339), and with ACMBFV isolates from Burkina Faso (HE616777, HE616779, HE616780, HE616781). The sequences of the 15 samples that tested positive for EACMCMV were most closely related to the EACMCMV and shared the highest nucleotide identities (97%–98%) with isolates from Ghana (MG250164), Ivory Coast (AF259896), Nigeria (EU685319, EU685326), and Madagascar (KJ887944). The ML phylogenetic tree inferred from alignment of coat protein (CP) gene sequences from Burkina Faso (25 ACMV‐like virus and six EACMCMV) and other CMGs confirmed that the sequences from Burkina Faso are phylogenetically associated with ACMV, ACMBFV, or EACMCMV (Figure 4).

## 4 DISCUSSION

In general, the mean CMD severity scores recorded in the study areas were moderate in both 2016 and 2017. This could be due to similarities in factors affecting disease establishment across the country. The significant difference observed between the proportion of localities with CMD‐affected fields in 2016 and 2017 could be explained by better awareness by farmers of the risk of CMD transmission via infected cuttings through outreach programmes initiated in 2016.

The relatively lower incidence recorded in Burkina Faso could be explained by the fact that the intensification of cassava production is a more recent phenomenon in the country compared to other African countries (Guira et al., 2017; Legg et al., 2006), coupled with CMD awareness and the adoption of good farming practices by farmers.

Our results showed that cases of CMD transmitted by cassava cuttings were more prevalent as compared to cases resulting from whitefly transmission. This phenomenon appears to be widespread in sub‐Saharan Africa (Chikoti et al., 2013; Mulenga et al., 2016; Mwatuni et al., 2015; Torkpo et al., 2018; Zinga et al., 2013). The high incidence of cutting‐borne infection is probably due to farmers’ inability to select virus‐free cassava cuttings when planting. The very low incidences of whitefly‐borne infections observed in Burkina Faso is consistent with the low counts of whiteflies observed in the cassava fields in both years under study. It is notable that although the mean whitefly counts in the Hauts‐Bassins region was less than one per plant in 2016, a higher proportion of whitefly‐borne infections were recorded from the region during the same period. These results can be interpreted as suggesting that the rate of whitefly‐borne infection is not always correlated with whitefly abundance, as was recently reported by Eni et al. (2021). Although our results showed that whiteflies may not be a key factor in the epidemiology of CMD in our study area, it would be interesting to conduct other field experiments using CMD‐free planting material, in different localities and at different times of the year, to determine the role played by whiteflies in CMD epidemiology.

This study shows the presence of ACMV‐like viruses in the eight cassava‐growing regions and EACMV‐like viruses in six of them, occurring as single or mixed infections in CMD‐affected cassava plants in Burkina Faso. This is probably due to the exchange of planting material between Burkina Faso and the neighbouring countries such as Ivory Coast, Togo, and Ghana where ACMV‐like viruses and EACMV‐like viruses have been also reported (Adjata et al., 2009; Torkpo et al., 2017; Toualy et al., 2014). ACMV‐like viruses were the predominant CMGs species in each cassava‐growing region as the majority of CMD resulted from single ACMV‐like virus infections. The predominance of single ACMV‐like virus infection in West Africa has previously been reported (Abubakar et al., 2019; Ogbe et al., 2006; Pita, Fondong, Sangaré, Kokora, et al., 2001; Toualy et al., 2014) accompanied by a low distribution of EACMV‐like virus single infections (Ariyo et al., 2005; Ogbe et al., 2006; Toualy et al., 2014). Our current work confirms that the situation has not changed. In addition, we discovered that most EACMV‐like virus isolates occurred as mixed infections with ACMV. Over 86% of the EACMV‐like virus positive samples were found to have East African cassava mosaic Cameroon virus (EACMCMV). These results show that EACMCMV occurrence may be more prevalent in Burkina Faso than previously thought. Our analysis confirms that the CMG isolates obtained from Burkina Faso samples are phylogenetically associated with ACMV‐like viruses (ACMV and ACMBFV) and EACMCMV. We propose further analysis, such as the use of specific primers for each CMG species or next‐generation sequencing, to resolve the issue of the occurrence of CMG species and strains in Burkina Faso.

We detected the occurrence of CMGs in symptomless samples (2.1%), which shows that the viruses can be latent in the plants without manifesting symptoms. Therefore, the use of symptomless cassava landraces as an option to manage CMD could inadvertently result in increased cutting‐borne transmission because they may harbour CMGs. We propose that the use of certified virus‐free cuttings for the establishment of new cassava fields will be crucial for fighting the transmission of CMD. In the absence of certified virus‐free cuttings, the training of farmers on how to select healthy cuttings for the new planting season and on use of in‐field diagnostic applications will be crucial to bring down the incidence or transmission of these viruses of high economic importance.

## CONFLICT OF INTEREST

The authors declare that they have no conflict of interest.

## Data Availability

The data that support the findings of this study are available from the corresponding author upon reasonable request.

## References

[ppa13459-bib-0001] Abubakar, M. , Singh, D. & Keta, J.N. (2019) Cassava mosaic disease and associated geminiviruses in Bauchi state, Nigeria: occurrence and distribution. American Journal of Plant Biology, 4, 85–90.

[ppa13459-bib-0002] Adjata, K.D. , Muller, E. , Peterschmi, M. , Traore, O. & Gumedzoe, Y.M.D. (2009) Molecular evidence for the association of a strain of Uganda variant of East African cassava mosaic virus to symptom severity in cassava (*Manihot esculenta* Crantz) fields in Togo. American Journal of Biochemistry and Biotechnology, 5, 196–201.

[ppa13459-bib-0003] Ally, H.M. , Hamss, H.E. , Simiand, C. , Maruthi, M.N. , Colvin, J. , Omongo, C.A. et al. (2019) What has changed in the outbreaking populations of the severe crop pest whitefly species in cassava in two decades? Scientific Reports, 9, 14796. 31615997 10.1038/s41598-019-50259-0 PMC6794263

[ppa13459-bib-0004] Ariyo, O.A. , Koerbler, M. , Dixon, A.G.O. , Atiri, G.I. & Winter, S. (2005) Molecular variability and distribution of cassava mosaic begomoviruses in Nigeria. Journal of Phytopathology, 153, 226–231.

[ppa13459-bib-0005] Bao, Y. , Chetvernin, V. & Tatusova, T. (2014) Improvements to pairwise sequence comparison (PASC): a genome‐based web tool for virus classification. Archives of Virology, 159, 3293–3304. 25119676 10.1007/s00705-014-2197-x PMC4221606

[ppa13459-bib-0006] Benjamini, Y. & Yekutieli, D. (2001) The control of the false discovery rate in multiple testing under dependency. Annals of Statistics, 29, 1165–1188.

[ppa13459-bib-0007] Berrie, L.C. , Rybicki, E.P. & Rey, M.E.C. (2001) Complete nucleotide sequence and host range of South African cassava mosaic virus: further evidence for recombination amongst begomoviruses. Journal of General Virology, 82, 53–58. 11125158 10.1099/0022-1317-82-1-53

[ppa13459-bib-0008] Bock, K.R. & Woods, R.D. (1983) Etiology of African cassava mosaic disease. Plant Disease, 67, 994–995.

[ppa13459-bib-0009] Bull, S.E. , Briddon, R.W. , Sserubombwe, W.S. , Ngugi, K. , Markham, P.G. & Stanley, J. (2006) Genetic diversity and phylogeography of cassava mosaic viruses in Kenya. Journal of General Virology, 87, 3053–3065. 16963765 10.1099/vir.0.82013-0

[ppa13459-bib-0010] Byju, G. & Suja, G. (2020) Mineral nutrition of cassava. Advances in Agronomy, 159, 169–235.

[ppa13459-bib-0011] Chikoti, P.C. , Ndunguru, J. , Melis, R. , Tairo, F. , Shanahan, P. & Sseruwagi, P. (2013) Cassava mosaic disease and associated viruses in Zambia: occurrence and distribution. International Journal of Pest Management, 59, 63–72.

[ppa13459-bib-0012] Dabiré, R. & Belem, J. (2003) Les plantes à tubercules et racines au Burkina Faso. WASNET, 8, 12–16.

[ppa13459-bib-0013] El‐Sharkawy, M.A. (2004) Cassava biology and physiology. Plant Molecular Biology, 56, 481–501. 15669146 10.1007/s11103-005-2270-7

[ppa13459-bib-0014] Eni, A.O. , Efekemo, O.P. , Onile‐ere, O.A. & Pita, J.S. (2021) South West and North Central Nigeria: assessment of cassava mosaic disease and field status of African cassava mosaic virus and East African cassava mosaic virus. Annals of Applied Biology, 178, 466–479. 34219746 10.1111/aab.12647 PMC8246719

[ppa13459-bib-0015] Fondong, V.N. , Pita, J.S. , Rey, M.E.C. , De Kochko, A. , Beachy, R.N. & Fauquet, C.M. (2000) Evidence of synergism between African cassava mosaic virus and a new double‐recombinant geminivirus infecting cassava in Cameroon. Journal of General Virology, 81, 287–297. 10640569 10.1099/0022-1317-81-1-287

[ppa13459-bib-0016] Guira, F. , Some, K. , Kabore, D. , Sawadogo‐Lingani, H. , Traore, Y. & Savadogo, A. (2017) Origins, production, and utilization of cassava in Burkina Faso, a contribution of a neglected crop to household food security. Food Science and Nutrition, 5, 415–423. 28572925 10.1002/fsn3.408 PMC5448348

[ppa13459-bib-0017] Konaté, G. , Barro, N. , Fargette, D. , Swanson, M.M. & Harrison, B.D. (1995) Occurrence of whitefly‐transmitted geminiviruses in crops in Burkina Faso and their serological detection and differentiation. Annals of Applied Biology, 126, 121–130.

[ppa13459-bib-0018] Kumar, S. , Stecher, G. , Li, M. , Knyaz, C. & Tamura, K. (2018) MEGA X: molecular evolutionary genetics analysis across computing platforms. Molecular Biology and Evolution, 35, 1547–1549. 29722887 10.1093/molbev/msy096 PMC5967553

[ppa13459-bib-0019] Legg, J.P. , French, R. , Rogan, D. , Okao‐Okuja, G. & Brown, J.K. (2002) A distinct *Bemisia tabaci* (Gennadius) (Hemiptera: Sternorrhyncha: Aleyrodidae) genotype cluster is associated with the epidemic of severe cassava mosaic virus disease in Uganda. Molecular Ecology, 11, 1219–1229. 12074729 10.1046/j.1365-294x.2002.01514.x

[ppa13459-bib-0020] Legg, J.P. , Owor, B. , Sseruwagi, P. & Ndunguru, J. (2006) Cassava mosaic virus disease in East and Central Africa: epidemiology and management of a regional pandemic. Advances in Virus Research, 67, 355–418. 17027685 10.1016/S0065-3527(06)67010-3

[ppa13459-bib-0021] MacFadyen, S. , Paull, C. , Boykin, L.M. , De Barro, P. , Maruthi, M.N. , Otim, M. et al. (2018) Cassava whitefly, *Bemisia tabaci* (Gennadius) (Hemiptera: Aleyrodidae) in East African farming landscapes: a review of the factors determining abundance. Bulletin of Entomological Research, 108, 565–582. 29433589 10.1017/S0007485318000032 PMC7672366

[ppa13459-bib-0022] Maruthi, M.N. , Seal, S. , Colvin, J. , Briddon, R.W. & Bull, S.E. (2004) East African cassava mosaic Zanzibar virus – a recombinant begomovirus species with a mild phenotype. Archives of Virology, 149, 2365–2377. 15375675 10.1007/s00705-004-0380-1

[ppa13459-bib-0023] Mugerwa, H. , Colvin, J. , Alicai, T. , Omongo, C.A. , Kabaalu, R. , Visendi, P. et al. (2021) Genetic diversity of whitefly (*Bemisia* spp.) on crop and uncultivated plants in Uganda: implications for the control of this devastating pest species complex in Africa. Journal of Pest Science, 94, 1307–1330. 34720787 10.1007/s10340-021-01355-6 PMC8550740

[ppa13459-bib-0024] Mulenga, R.M. , Legg, J.P. , Ndunguru, J. , Miano, D.W. , Mutitu, E.W. , Chikoti, P.C. et al. (2016) Survey, molecular detection, and characterization of geminiviruses associated with cassava mosaic disease in Zambia. Plant Disease, 100, 1379–1387. 30686191 10.1094/PDIS-10-15-1170-RE

[ppa13459-bib-0025] Mwatuni, F. , Ateka, E. , Karanja, L. , Mwaura, S. & Obare, I. (2015) Distribution of cassava mosaic geminiviruses and their associated DNA satellites in Kenya. American Journal of Experimental Agriculture, 9, 1–12.

[ppa13459-bib-0026] Ntawuruhunga, P. , Dixon, A.G.O. , Kanju, E. , Ssemakula, G. , Okechukwu, R.U. , Whyte, J.B.A. et al. (2013) Successful innovations and lessons learnt in cassava improvement and deployment by IITA in the Eastern African Region. African Journal of Root and Tuber Crops, 10, 41–54.

[ppa13459-bib-0027] Ogbe, F.O. , Dixon, A.G.O. , Hughes, J.D'A. , Alabi, O.J. & Okechukwu, R. (2006) Status of cassava begomoviruses and their new natural hosts in Nigeria. Plant Disease, 90, 548–553. 30781126 10.1094/PD-90-0548

[ppa13459-bib-0028] Patil, B.L. & Fauquet, C.M. (2009) Cassava mosaic geminiviruses: actual knowledge and perspectives. Molecular Plant Pathology, 10, 685–701. 19694957 10.1111/j.1364-3703.2009.00559.x PMC6640248

[ppa13459-bib-0029] Permingeat, H.R. , Romagnoli, M.V. , Juliana, I. & Vallejos, R.H. (1998) A simple method for isolating DNA of high yield and quality from cotton (*Gossypium hirsutum* L.) leaves. Plant Molecular Biology Reporter, 16, 89.

[ppa13459-bib-0030] Pita, J.S. , Fondong, V.N. , Sangaré, A. , Kokora, R.N.N. & Fauquet, C.M. (2001) Genomic and biological diversity of the African cassava geminiviruses. Euphytica, 120, 115–125.

[ppa13459-bib-0031] Pita, J.S. , Fondong, V.N. , Sangaré, A. , Otim‐Nape, G.W. , Ogwal, S. & Fauquet, C.M. (2001) Recombination, pseudorecombination and synergism of geminiviruses are determinant keys to the epidemic of severe cassava mosaic disease in Uganda. Journal of General Virology, 82, 655–665. 11172108 10.1099/0022-1317-82-3-655

[ppa13459-bib-0032] Price, M.N. , Dehal, P.S. & Arkin, A.P. (2010) FastTree 2 – approximately maximum‐likelihood trees for large alignments. PLoS One, 5, e9490. 20224823 10.1371/journal.pone.0009490 PMC2835736

[ppa13459-bib-0033] Pushpalatha, R. & Gangadharan, B. (2020) Is cassava (*Manihot esculenta* Crantz) a climate “smart” crop? A review in the context of bridging future food demand gap. Tropical Plant Biology, 13, 201–211.

[ppa13459-bib-0034] Saediman, H. , Limi, M.A. , Rosmawaty, A.P. & Indarsyih, Y. (2016) Cassava consumption and food security status among cassava growing households in southeast Sulawesi. Pakistan Journal of Nutrition, 15, 1008–1016.

[ppa13459-bib-0035] Sseruwagi, P. , Sserubombwe, W.S. , Legg, J.P. , Ndunguru, J. & Thresh, J.M. (2004) Methods of surveying the incidence and severity of cassava mosaic disease and whitefly vector populations on cassava in Africa: a review. Virus Research, 100, 129–142. 15036844 10.1016/j.virusres.2003.12.021

[ppa13459-bib-0036] Stanley, J. & Gay, M. (1983) Nucleotide sequence of cassava latent virus DNA. Nature, 301, 260–262.

[ppa13459-bib-0037] Terry, E.R. (1975) Description and evaluation of cassava mosaic disease in Africa. In: Terry, E.R. & MacIntyre, R. (Eds.) The international exchange and testing of cassava germplasm in Africa. Ibadan, Nigeria: IITA, pp. 53–54.

[ppa13459-bib-0038] Tiendrébéogo, F. , Lefeuvre, P. , Hoareau, M. , Harimalala, M.A. , De Bruyn, A. , Villemot, J. et al. (2012) Evolution of African cassava mosaic virus by recombination between bipartite and monopartite begomoviruses. Virology Journal, 9, 1–7. 22416906 10.1186/1743-422X-9-67 PMC3328289

[ppa13459-bib-0039] Tiendrébéogo, F. , Lefeuvre, P. , Hoareau, M. , Traoré, V.S.E. , Barro, N. , Reynaud, B. et al. (2009) Occurrence of East African cassava mosaic virus ‐Uganda (EACMV‐UG) in Burkina Faso. Plant Pathology, 58, 783.

[ppa13459-bib-0040] Torkpo, S.K. , Gafni, Y. , Danquah, E.Y. & Offei, S.K. (2018) Incidence and severity of cassava mosaic disease in farmer’s fields in Ghana. Ghana Journal of Agricultural Science, 53, 61.

[ppa13459-bib-0041] Torkpo, S.K. , Offei, K. , Danquah, E.Y. & Gafni, Y. (2017) Status of cassava mosaic begomoviruses in farmers’ fields in Ghana. AIMS Agriculture and Food, 2, 279–289.

[ppa13459-bib-0042] Toualy, M.N.Y. , Akinbade, S.A. , Koutoua, S. , Diallo, H.A. & Lava, P.K. (2014) Incidence and distribution of cassava mosaic begomoviruses in Côte d’Ivoire. International Journal of Agronomy and Agricultural Research, 4, 131–139.

[ppa13459-bib-0043] Zhou, X. , Robinson, D.J. & Harrison, B.D. (1998) Types of variation in DNA‐A among isolates of East African cassava mosaic virus from Kenya, Malawi and Tanzania. Journal of General Virology, 79, 2835–2840. 9820161 10.1099/0022-1317-79-11-2835

[ppa13459-bib-0044] Zinga, I. , Chiroleu, F. , Legg, J. , Lefeuvre, P. , Komba, E.K. , Semballa, S. et al. (2013) Epidemiological assessment of cassava mosaic disease in Central African Republic reveals the importance of mixed viral infection and poor health of plant cuttings. Crop Protection, 44, 6–12.

